# Editorial: Immunotherapy: up to date progress in childhood and hematological malignancies

**DOI:** 10.3389/fonc.2025.1584101

**Published:** 2025-03-27

**Authors:** Kishore B. Challagundla, Kausar M. Ansari, Manoj K. Pandey

**Affiliations:** ^1^ Department of Basic Biomedical Sciences, Touro College of Osteopathic Medicine, Middletown, NY, United States; ^2^ Food, Drug & Chemical Toxicology, Council of Scientific and Industrial Research (CSIR)-Indian Institute of Toxicology Research, Lucknow, Uttar Pradesh, India; ^3^ Department of Biomedical Sciences, Cooper Medical School of Rowan University, Camden, NJ, United States

**Keywords:** immunotherapy, childhood malignancies, hematological malignancies, resistance, CAR-T, immune activation

## Background

A majority of immunotherapy studies have focused on adult malignancies. Nevertheless, numerous medications have demonstrated potential efficacy in pediatric populations. Childhood hematologic malignancies are very infrequent although are reported to be a major cause of death in children aged 1-14. Immunotherapy has become an important aspect of treatment for many tumors. The area is continually changing as novel immunotherapy therapies are being evaluated and approved and new techniques of working with the immune system are being found at a fast pace. There are a range of immunotherapies and the development of undesired side effects as well as resistance. Developments in the study of tumor immunology have resulted in fresh information which welcomed the development of tumor-associated antigens for immune cell targeting. There has been a significant boom in immune-based therapy for childhood hematologic malignancies and how we treat children with these tumors in general. This Research Topic brings together leading-edge research that underlines the important role of immunotherapy in modern pediatric oncology. We anticipate our findings will contribute to the ongoing clinical translation of innovative immunotherapies, ultimately enhancing patient outcomes and quality of life in pediatric and hematological cancers.

The first review addresses the effect of immunotherapy in Chronic Active Epstein-Barr Virus Disease (CAEBV), a severe lymphoproliferative illness caused by EBV infection in T and NK cells leading to immune system failure and an increased risk of lymphoma. While hematopoietic stem cell transplantation (HSCT) remains the primary curative treatment, current breakthroughs in immunotherapy provide hopeful alternatives. Checkpoint inhibitors such as PD-1 blockers (Nivolumab, Sintilimab) increase immune responses against EBV-infected cells, whereas bispecific antibodies (gp350/CD89 targeting) promote immune-mediated clearance. Cell-based therapies are also emerging as effective treatments, including EBV-specific cytotoxic T lymphocytes (EBV-CTLs) to restore immunological control, CAR-T therapies targeting gp350 and LMP2A to remove infected cells, and TCR-T cells designed to increase T-cell identification of EBV antigens. Although HSCT remains the gold standard, immunotherapies including CAR-T, checkpoint inhibitors, and EBV-CTLs are altering CAEBV treatment. Further research is necessary to integrate these medicines into clinical practice, enhancing survival rates and decreasing treatment-related harm.

The second research article examines the influence of disease burden and late onset of B-cell aplasia (BCA) on the risk of relapse after CD19 chimeric antigen receptor (CAR) T-cell therapy (Tisagenlecleucel) in pediatric and young adult patients with relapsed/refractory B-cell acute lymphoblastic leukemia (R/R B-ALL). The research, carried out in various Spanish hospitals, examined 73 individuals and classified them into high tumor burden (HTB) and low tumor burden (LTB) categories. Results indicated that HTB patients had a markedly reduced 12-month event-free survival (EFS) of 19.3%, in contrast to 67.2% in LTB patients. Relapse patterns differed, with HTB patients primarily encountering CD19-negative relapses (72%) and LTB patients facing CD19-positive relapses (71%), frequently preceded by the loss of BCA. The delayed elimination of BCA beyond six months post-infusion correlated with an increased likelihood of CD19-positive recurrence, underscoring the need for ongoing immunological surveillance. The research highlights the significance of BCA monitoring as an indicator of relapse, especially in LTB patients, and proposes early intervention methods such allogeneic stem cell transplantation (allo-SCT) for HTB patients. Standardizing BCA monitoring procedures may improve relapse prediction and refine post-CAR-T treatment options.

The third case report outlines the effective application of CD19 and CD20 monoclonal antibody therapy in conjunction with chemotherapy for a 4-year-old patient with refractory B-cell acute lymphoblastic leukemia (B-ALL). Notwithstanding normal induction therapy, the patient exhibited enduring minimum residual disease (MRD) and a high-risk prognosis. To improve disease management, the treatment team integrated Blinatumomab (CD19-targeting) and Rituximab (CD20-targeting) with three cycles of consolidation chemotherapy. This innovative method resulted in MRD clearance, allowing the youngster to successfully receive allogeneic hematopoietic stem cell transplantation (allo-HSCT). The patient tolerated the monoclonal antibody treatment effectively, exhibiting no notable neurotoxicity or cytokine release syndrome (CRS). During the follow-up, the youngster sustained complete remission for more than 13 months, illustrating the effectiveness and safety of this sequential immunotherapy approach. This case represents the inaugural recorded application of CD20-targeted therapy in pediatric B-ALL, indicating that the integration of dual monoclonal antibodies may augment leukemia treatment regimens and enhance survival rates. Nevertheless, additional clinical investigations are required to confirm the long-term advantages of this method.

The fourth study article examines the influence of pre-infusion tumor load and subsequent loss of B-cell aplasia (BCA) on the likelihood of relapse in pediatric and young adult patients with relapsed/refractory B-cell acute lymphoblastic leukemia (R/R B-ALL) after CD19 CAR-T treatment (Tisagenlecleucel). The research, derived from a Spanish multicenter retrospective analysis, classifies patients into high tumor burden (HTB) and low tumor burden (LTB) cohorts and investigates their event-free survival (EFS) and relapse trends. The results demonstrate that HTB patients have markedly poorer outcomes, exhibiting a 12-month event-free survival rate of 19.3%, in contrast to 67.2% for LTB patients. HTB patients generally relapse with CD19-negative illness (72%), while LTB patients often have CD19-positive relapse (71%), regularly preceded by the loss of BCA. The research demonstrates a significant association between BCA loss and CD19-positive recurrence, especially when it occurs after six months post-infusion, highlighting the necessity for prolonged monitoring and possible therapeutic measures. The research indicates that HTB patients must to be evaluated for early allogeneic stem cell transplantation (allo-SCT), but LTB patients experiencing late BCA loss necessitate rigorous monitoring of minimal residual disease (MRD). Standardizing BCA monitoring techniques may augment relapse prediction and refine post-CAR-T treatment options for pediatric leukemia patients.

The concluding review article “*The Immunotherapy Advancement Targeting Malignant Blastomas in Early Childhood*” examines current advancements in immunotherapy for the treatment of childhood blastomas, including neuroblastoma, retinoblastoma, hepatoblastoma, pleuropulmonary blastoma, and pancreatic blastoma. Conventional therapies such as surgery, chemotherapy, and radiotherapy possess limitations, leading to increased interest in monoclonal antibodies and chimeric antigen receptor (CAR) T-cell therapy. Treatment for neuroblastoma has progressed with the use of anti-GD2 monoclonal antibodies such Dinutuximab, however toxicity continues to be a concern. Research on retinoblastoma emphasizes GD2-CAR T cells, demonstrating potential in preclinical studies. Therapies for hepatoblastoma focus on Glypican-3 (GPC3) through the utilization of CAR-T cells and peptide vaccines. Although CAR-T therapy has demonstrated efficacy in leukemia, its utilization in solid tumors such as blastomas encounters obstacles due to antigen evasion and the tumor microenvironment. Notwithstanding these obstacles, immunotherapy is emerging as a revolutionary method, providing focused and less harmful options. Additional clinical trials are required to refine treatment options and include immunotherapy into conventional care for juvenile blastomas.

## Conclusions

This Research Topic presents research that collectively emphasize the transformative potential of immunotherapy in the treatment of pediatric and hematological cancers. Principal discoveries indicate that: Checkpoint inhibitors and CAR-T treatments are broadening therapeutic alternatives for EBV-related illnesses, B-ALL, and blastomas. Monitoring immunological responses, such as BCA loss and MRD status, is essential for forecasting relapse risks and enhancing post-CAR-T treatments. Combination immunotherapy strategies, such as the use of CD19/CD20 antibodies alongside chemotherapy, can enhance long-term outcomes and facilitate access to allogeneic hematopoietic stem cell transplantation (HSCT). Ongoing clinical trials are essential to confirm the safety, efficacy, and feasibility of novel immunotherapies within standard pediatric oncology regimens.

## Future directions

The evolution of pediatric and hematologic oncology necessitates the integration of personalized immunotherapy, early immune surveillance, and combination treatment strategies to improve survival rates and minimize treatment-related toxicities. Future research should concentrate on refining CAR-T therapy designs to address antigen escape mechanisms and enhance long-term persistence. Investigating bispecific antibodies and immune-modulating agents to improve anti-tumor responses. Developing standardized tools for monitoring tumor burden and tracking immune function. Optimizing combination therapies for pediatric cancers that are high-risk and relapsed. Advancing our understanding of immune-based interventions may lead to curative and less toxic treatment options for children and young adults with hematological and solid malignancies.

